# *BRAF* Mutations Classes I, II, and III in NSCLC Patients Included in the SLLIP Trial: The Need for a New Pre-Clinical Treatment Rationale

**DOI:** 10.3390/cancers11091381

**Published:** 2019-09-17

**Authors:** Jillian Wilhelmina Paulina Bracht, Niki Karachaliou, Trever Bivona, Richard B. Lanman, Iris Faull, Rebecca J. Nagy, Ana Drozdowskyj, Jordi Berenguer, Manuel Fernandez-Bruno, Miguel Angel Molina-Vila, Rafael Rosell

**Affiliations:** 1 Pangaea Oncology, Laboratory of Molecular Biology, Quirón-Dexeus University Institute, 08028 Barcelona, Spain; 2 Department of Biochemistry, Molecular Biology and Biomedicine, Universitat Autònoma de Barcelona (UAB), 08193 Bellaterra (Barcelona), Spain; 3 Instituto Oncológico Rosell (IOR), University Hospital Sagrat Cor, QuironSalud Group, 08029 Barcelona, Spain; 4 Department of Medicine, University of California, San Francisco, CA 94143, USA; 5 Guardant Health, Inc., Redwood City, CA 94063, USA; 6 PIVOTAL SL, 28023 Madrid, Spain; 7 Institute for Health Science Research Germans Trias i Pujol (IGTP), 08916 Badalona, Spain

**Keywords:** BRAF, NSCLC, SHP2, PTPN11, MEK, MAPK

## Abstract

*BRAF* V600 mutations have been found in 1–2% of non-small-cell lung cancer (NSCLC) patients, with Food and Drug Administration (FDA) approved treatment of dabrafenib plus trametinib and progression free survival (PFS) of 10.9 months. However, 50–80% of *BRAF* mutations in lung cancer are non-V600, and can be class II, with intermediate to high kinase activity and RAS independence, or class III, with impaired kinase activity, upstream signaling dependence, and consequently, sensitivity to receptor tyrosine kinase (RTK) inhibitors. Plasma cell-free DNA (cfDNA) of 185 newly diagnosed advanced lung adenocarcinoma patients (Spanish Lung Liquid versus Invasive Biopsy Program, SLLIP, NCT03248089) was examined for *BRAF* and other alterations with a targeted cfDNA next-generation sequencing (NGS) assay (Guardant360®, Guardant Health Inc., CA, USA), and results were correlated with patient outcome. Cell viability with single or combined RAF, MEK, and SHP2 inhibitors was assessed in cell lines with *BRAF* class I, II, and III mutations. Out of 185 patients, 22 had *BRAF* alterations (12%) of which seven patients harbored amplifications (32%) and 17 had *BRAF* mutations (77%). Of the *BRAF* mutations, four out of 22 (18%) were V600E and 18/22 (82%) were non-V600. In vitro results confirmed sensitivity of class III and resistance of class I and II *BRAF* mutations, and BRAF wild type cells to SHP2 inhibition. Concomitant MEK or RAF and SHP2 inhibition showed synergistic effects, especially in the class III *BRAF*-mutant cell line. Our study indicates that the class of the *BRAF* mutation may have clinical implications and therefore should be defined in the clinical practice and used to guide therapeutic decisions.

## 1. Introduction

BRAF is a member of the rapidly accelerated fibrosarcoma (RAF) kinase family, which transduces signals downstream of RAS via the mitogen-activated protein kinase (MAPK) pathway and induces cell growth and survival [1]. Signals derived from activated receptor tyrosine kinases (RTKs) control the activation of RAS proteins through son-of-sevenless (SOS) and neurofibromin 1 (NF1), which activate and inactivate RAS, respectively. RAS can consequently stimulate RAF kinases through hetero- or homodimer formation, which in turn leads to MEK and ERK phosphorylation and activation. Under physiologic conditions, the MAPK pathway is tightly regulated through negative feedback loops. However, mutations in *BRAF* can independently lead to uncontrolled cellular proliferation and cell survival through ERK signaling, and have been detected in melanoma (50–60%), colorectal cancer (10%), thyroid cancer (30–50%), serous ovarian cancer (30%), and non-small-cell lung cancer (NSCLC; 3%), amongst others [2,3]. Although these tumor types are located in different organs, it could be hypothesized that the functional consequence, and therefore the effect of treatment, of these mutations are similar. 

In NSCLC, *BRAF* V600 mutations have been found in 1–2% of patients, and are categorized as class I *BRAF* mutations [4]. Dual MAPK pathway inhibition using dabrafenib (BRAF inhibitor) plus trametinib (MEK inhibitor) achieved a 64% response rate and a median progression-free survival (PFS) of 10.9 months in *BRAF* V600 mutation-positive NSCLC [5], and the combined treatment was approved by the Food and Drug Administration (FDA) in 2017 [6]. Effectivity of combined MEK and BRAF inhibition can be explained by the functional effect of class I *BRAF* mutations. *BRAF* V600D/E/K/R mutations result in a strong activation of the BRAF kinase and the MAPK pathway, while being RAS-independent. In fact, RAS activation is even suppressed in class I *BRAF* mutations, due to a negative feedback loop after ERK activation [1]. 

Next-generation sequencing (NGS) and other technical improvements have revealed that 50%-80% of *BRAF* alterations in lung cancer are non-V600 and have considerably different signaling properties [1,7]. Non-V600 *BRAF* mutations can be subdivided into class II, with intermediate to high kinase activity and RAS independence, class III, with a lack of or impaired kinase activity, and “other” *BRAF* mutations that have not been classified [1,4,8,9]. In addition, class III mutations harbor *RAS* activating mutations, *NF1* tumor suppressor deletions, or depend on upstream receptor tyrosine kinase (RTK) signaling for cell growth [7]. This dependence suggests that class III *BRAF* mutant tumors are sensitive to RAS inhibition using RTK inhibitors [10]. However, so far there are no effective targeted treatments available for patients harboring *KRAS*, *NF1*, and non-V600 *BRAF* alterations. 

The non-receptor protein tyrosine phosphatase and scaffold protein SHP2 (*PTPN11*) functions downstream of multiple RTKs, and promotes RAS activation after induction by growth factor signals [7]. *SHP2* mutations can consequently drive RAS/MAPK signaling, independent of RTK activation. Inhibiting SHP2 activity has been shown to suppress tumor cell growth by decreasing RAS/MAPK signaling, specifically in RTK-dependent cells [11]. Since SHP2 comprises a convergent point in between RTK and downstream signaling, it has potential to be used as a new therapeutic target for cancer therapy. Different SHP2 inhibitors have been discovered and tested. SHP099 is a selective small-molecule inhibitor and stabilizes SHP2 through allosteric inhibition. In vitro and in vivo models have shown that SHP099 inhibits RAS/MAPK signaling in RTK driven cancer cells and suppresses cell proliferation. RMC-4550 is a more potent and selective allosteric inhibitor of SHP2, and was initially developed to treat esophageal tumors with *EGFR* driver mutations [12]. 

Based on the signaling mechanisms of the different *BRAF* mutant classes, only class III *BRAF* mutations are expected to be sensitive to single SHP2 inhibition due to the dependence of this specific *BRAF* mutation on upstream signaling. Moreover, *KRAS* G12C mutations and *NF1* loss-of-function mutations confer sensitivity to SHP2 inhibition [7]. Bearing in mind that one in five NSCLC patients harbor one of these driver mutations, combining SHP2 inhibitors with BRAF or MEK inhibitors could have a huge impact on clinical outcome. Since class II *BRAF* mutations are not RAS-dependent, it is unlikely that targeting SHP2 will affect cell proliferation. However, combining SHP2 with MAPK pathway inhibitors may induce synthetic lethality in this class of *BRAF* mutations. 

In the Spanish Lung Liquid versus Invasive Biopsy Program (SLLIP) study, genomic profiling was performed on plasma samples of 185 treatment-naïve advanced lung adenocarcinoma patients, using a 73-gene cell-free DNA (cfDNA) assay (Guardant360) (NCT03248089). A secondary aim of the study was the discovery of additional drivers and actionable mutations in plasma, particularly in the absence of *EGFR*, *KRAS*, *ALK*, and *ROS1* alterations [13].

In this study, we report on the frequency of different *BRAF* mutation classes that were detected in the cfDNA of patients from the SLLIP study and correlate the mutation classes with clinical outcome. In addition, we use in vitro studies in three distinct *BRAF*-mutant cell lines of different origin to highlight the relevance and importance of specific targeted treatment based on *BRAF* mutation class. 

## 2. Results

### 2.1. Different Classes of BRAF Mutations and Co-Alterations in Cell-Free DNA of Lung Cancer Patients

**Figure 1:**
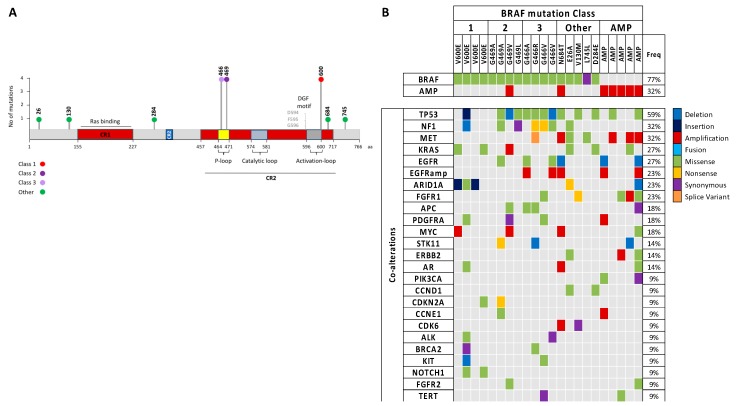
(**A**) Lollipop plot from the plasma cfDNA sequencing results, representing the classification of the detected *BRAF* mutations (*n* = 17). Class I, II, and III mutations are indicated in red, purple, and lilac, respectively, and “other” mutations are indicated in green. Amplifications are not depicted in this plot; (**B**) OncoPrint representing the most common co-alterations detected in the *BRAF*-alteration positive cohort. Individual samples are represented as columns and grouped by *BRAF* class, while individual genes are represented as rows. The alterations are represented as follows: deletion (blue), insertion (navy blue), amplification (red), fusion (turquoise), missense mutation (green), nonsense mutation (yellow), synonymous mutation (purple), and splice variant (orange). cfDNA: cell-free DNA; AMP: amplification; Freq: frequency.

From August 2016 to June 2017, the plasma cfDNA of 185 newly diagnosed, treatment naïve, advanced lung adenocarcinoma patients was examined for alterations in 73 genes using NGS. *BRAF* mutations were found in 17 (9%), and *BRAF* amplifications in seven (4%) patients, of which two patients also harbored a *BRAF* mutation, as shown in Figure 1A and Table 1. Out of these 22 *BRAF*-alteration positive patients, class I *BRAF* mutations were detected in four patients (18%). Non-V600 *BRAF* alterations were found in the other 18 (82%) patients, including seven patients with amplifications, four patients harboring class II (G469V/A/L), and four more class III (G466V/R/A) mutations, the latter two located in the P-loop of the *BRAF* kinase domain. The mutations that could not be classified into class I, II, or III are depicted as “other” mutations in the lollipop plot of the *BRAF* sequence, as shown in Figure 1A. 

Hereafter, co-alterations in the other genes of the panel were explored, as shown in Figure 1B. The main co-occurring alterations in the class I *BRAF*-mutant patients were AT-rich interactive domain-containing protein 1A (*ARID1A*) mutations in three of them, two *KRAS* mutations, and two mutations in Notch homolog 1, translocation-associated (Drosophila) (*NOTCH1*). In the non-V600 *BRAF*-mutant group, co-occurring mutations in *TP53* were found in most patients (67%), indicating the need for dual treatment with compounds that can restore the *TP53* function. Class III *BRAF* mutations were shown to have a lack of—or impaired—kinase activity and depend on *RAS* activating mutations, *NF1* deletions, or upstream RTK signaling for cell growth [7]. *NF1* loss of function mutations can cause constitutively active RAS signaling, and were found in three out of four class III *BRAF* mutation-positive patients, in addition to three *EGFR* alterations.

### 2.2. BRAF Class Correlated to Progression-Free Survival

**Figure 2:**
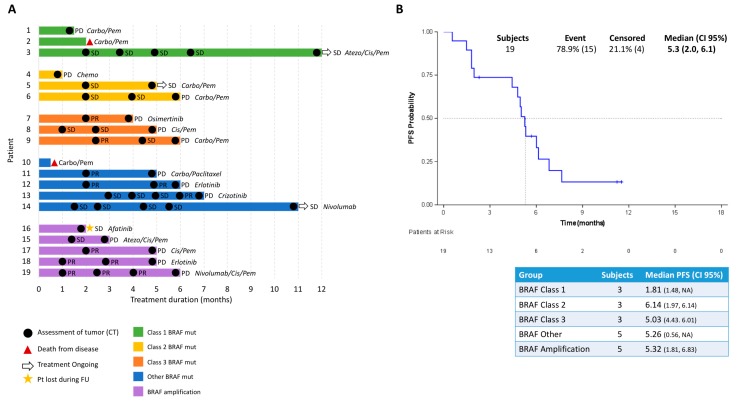
(**A**) Swimmer’s plot of time (months) on first-line treatment with clinical response for evaluable patients (*n* = 19). Clinical response is represented as follows: progressive disease (PD), stable disease (SD), partial response (PR), and complete response (CR); (**B**) Kaplan–Meier curve of progression free survival (PFS) among all *BRAF*-alteration positive patients. Table indicates median PFS in each *BRAF* class. Carbo: carboplatin; Pem: pemetrexed; Atezo: atezolizumab; Cis: cisplatin; Chemo: chemotherapy; Pt: patient; FU: follow-up.

In the SLLIP trial, patients were treated with chemotherapy and/or immunotherapy, or targeted therapies where applicable (see Table 1). To explore the effect of *BRAF* mutation class on treatment outcome, we correlated *BRAF* class to PFS of patients. Three patients did not receive treatment and were excluded from the analysis. Swimmer plot visualization, as shown in Figure 2A, indicates treatment duration, assessment of CT scans according to RECIST criteria, and if treatment was continued after 12 months of follow-up. 

The Kaplan–Meier estimator of the *BRAF*-alteration positive patients, as shown in Figure 2B, illustrates a median PFS of only 5.3 months (confidence interval 95% [CI 95%]: 2.0–6.1 months). Separating patients into classes, although the number of patients is small, hints at lower PFS in the class I *BRAF*-mutant patient group, with a median of 1.8 months. Class II and III *BRAF*-mutant patients had a median PFS of 6.1 and 5.0 months, respectively. The “other” *BRAF*-mutant patients and the *BRAF* amplified cohort displayed a median PFS of 5.3 months. These results are based on small cohorts but suggest that treatment outcomes may differ amongst patients with diverse *BRAF* alterations, and adequate therapies may influence treatment response for specific *BRAF* classes. 

### 2.3. Distinct BRAF Classes Require Specific Drug Combinations In Vitro

**Figure 3:**
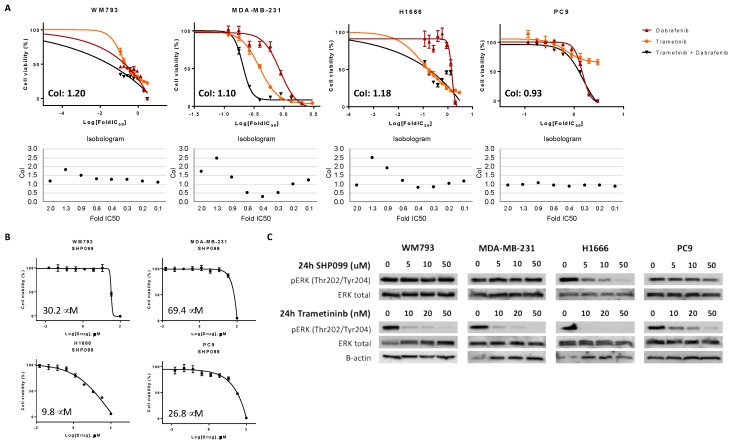
(**A**) Cell viability assays were performed to compare the effect of single MEK (trametinib), single BRAF (dabrafenib), or combined treatment on cell viability in the class I *BRAF*-mutant WM793 cell line, the class II *BRAF*-mutant MDA-MB-231 cell line, the class III *BRAF*-mutant H1666 cell line, and in the *BRAF*-WT PC9 cell line. The isobolograms depict combination index (CoI) values at each drug concentration, calculated based on the Chou and Talalay method. Average CoIs are depicted in the graph, and CoI values <1, = 1, and >1 indicate synergism, additive effect, and antagonism, respectively. Experiments were performed in biological triplicates with similar results, and representative graphs are shown. (**B**) Cell viability assays were performed to determine the half maximal inhibitory concentration (IC_50_) of SHP099 in each cell line. IC_50s_ are presented in the graphs. Experiments were performed in biological triplicates with similar results, and representative graphs are shown. **(C)** Cell lines were treated with single SHP099 (5, 10, and 50 μM), or single trametinib (10, 20, and 50 nM). Untreated cells received an equivalent dose of vehicle (DMSO). Cell lysates were used for immunoblotting and changes in ERK and phosphorylated ERK (Thr202/Tyr204) upon the different treatments were investigated. Experiments were performed in biological triplicates with similar results, and a representative blot is shown.

While class I *BRAF* mutations can now be effectively treated with the combination of dabrafenib and trametinib due to strong activation of the BRAF kinase and RAS signaling independence [5], non-V600 *BRAF* mutations have considerably different signaling properties [1] and therefore require other targeted treatments. To determine the effect of combined dabrafenib and trametinib on cell viability, we treated four distinct cell lines harboring either (1) the class I V600E *BRAF* mutation, (2) the class II non-V600 *BRAF* mutation, (3) the class III non-V600 *BRAF* mutation, or (4) *BRAF* wild type (WT), as shown in Table 2. Figure 3A indicates that cell viability assays with dual dabrafenib and trametinib treatment has distinct effects in cell lines with different *BRAF* classes. While the effects in class I, III, and *BRAF* WT cells were additive to antagonistic, combined treatment showed synergism at intermediate concentrations in the MDA-MB-231 cell line harboring a class II *BRAF* mutation. 

Since certain classes of *BRAF* mutations—or additional co-alterations—confer sensitivity to SHP2 inhibition, we aimed to determine the effect of SHP2 inhibition in the cell lines by measuring the half maximal inhibitory concentrations (IC_50s_) after treatment with SHP099 (all cell lines) and RMC-4550 (class II and class III cell lines) using cell viability assays. As expected, the class III *BRAF*-mutant cell line H1666 was sensitive to single SHP2 inhibition with SHP099, with an IC_50_ of 9.8 μM, as shown in Table 2 and Figure 3B. In contrast, the class II *BRAF*-mutant cell line showed resistance to the same treatment, with an IC_50_ of 69.4 μM. Both the class I and *BRAF* WT cell lines were intermediately sensitive to SHP2 inhibition compared to the other cell lines, with IC_50s_ of 30.2 and 26.8 μM, respectively. A different SHP2 inhibitor, RMC-4550, showed similar results in the class II and class III *BRAF*-mutant cell lines, with slightly lower IC_50s_ of 63.0 μM in the class II and 3.1 μM in the class III *BRAF*-mutant cell line, as shown in Table 2 and Figure S1. Hereafter, we checked the effect of different concentrations of single SHP2 and single MEK inhibition on downstream ERK activation using immunoblotting experiments. Figure 3C indicates that single trametinib treatment can completely inhibit phosphorylation of ERK at Thr202/Tyr204, in all *BRAF* mutant cell lines. In the PC9 cell line, a higher concentration of trametinib is needed to inhibit ERK activation, consistent with the lack of alterations—and therefore sensitivity to inhibition—in the MAPK pathway. Treatment with increasing doses of SHP099 reveals that ERK phosphorylation is not inhibited in the class I, II, and WT cell line, and therefore explains that single SHP2 inhibition is not effective enough to inhibit cell viability. In contrast, SHP099 inhibits ERK phosphorylation in the class III *BRAF* mutant cell line H1666. This finding explains why this cell line is more sensitive to cell growth inhibition after single SHP2 treatment. 

**Figure 4:**
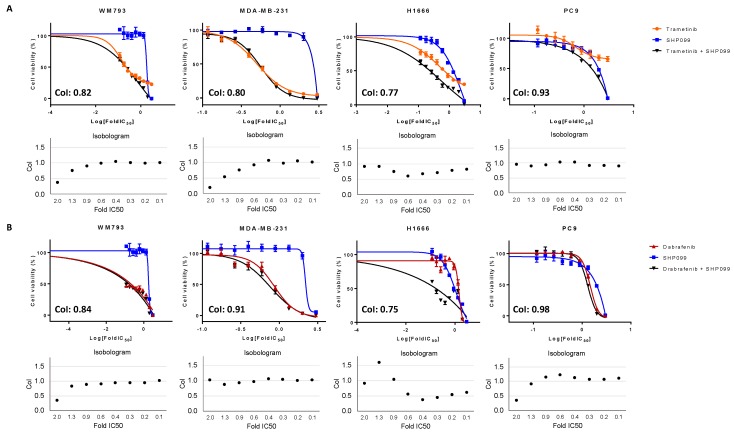
MTT cell viability assays were performed in the class I (WM793), class II (MDA-MB-231), and class III (H1666) *BRAF*-mutant cell lines and one BRAF WT cell line (PC9), to compare the effect of single MEK (trametinib) and single SHP099, or combined treatment on cell viability (**A**) and to compare the effect of single BRAF (dabrafenib) and single SHP099, or combined treatment on cell viability (**B**). Isobolograms depict combination index (CoI) values at each drug concentration, calculated based on the Chou and Talalay method. Average CoIs are depicted in the graph, and CoI values <1, = 1, and >1 indicate synergism, additive effect, and antagonism, respectively. Experiments were performed in biological triplicates with similar results, and representative graphs are shown.

Currently, no effective targeted treatments are available for patients harboring *KRAS, NF1*, and non-V600 *BRAF* alterations. Due to the potent effect of SHP2 inhibition on cell viability in the class III *BRAF*-mutant cell line, we hypothesized that combined MAPK and SHP2 inhibition may enhance the inhibitory effect on cell viability in non-V600 *BRAF*-mutant cell lines. Therefore, combined SHP2 and BRAF or MEK inhibitors were tested in the class I, II, and III *BRAF*-mutant cell lines and the BRAF WT cell line. In the class I (WM793) and II (MDA-MB-231) *BRAF*-mutant cell lines, combining MEK or BRAF with SHP2 inhibition yielded average CoIs indicative of moderate synergistic effects. However, as expected from previously found resistance to SHP2 inhibition, this effect is mainly due to dabrafenib or trametinib treatment, as shown in Figure 4A,B. For the class I cell line, CoIs of SHP099 combined with trametinib or dabrafenib were 0.82 and 0.84, respectively. The class II cell line displayed CoIs of 0.80 and 0.91 for combined treatment of trametinib or dabrafenib with SHP099, as shown in Figure 4A,B, and 0.91 and 0.92 for combined treatment of trametinib or dabrafenib with RMC-4550, as shown in Figure S2A,B. Similar results can be seen in the *BRAF* WT cell line (PC9), where CoI values are indicative of additive effects from the combined drugs reaching 0.98 for combined dabrafenib and SHP099 and 0.93 for the combination of trametinib and SHP099. While the combination of trametinib and dabrafenib in the class III *BRAF*-mutant cell line (H1666) showed antagonistic effects, combining BRAF or MEK inhibitors with either SHP099, as shown in Figure 4A,B, or RMC-4550, as shown in Figure S2A,B, yielded synergistic results. Average CoIs of SHP099 were 0.77 when combined with trametinib, and 0.75 when combined with dabrafenib. For RMC-4550, CoIs were 0.67 with trametinib, and 0.90 when combined with dabrafenib treatment.

## 3. Discussion

**Figure 5:**
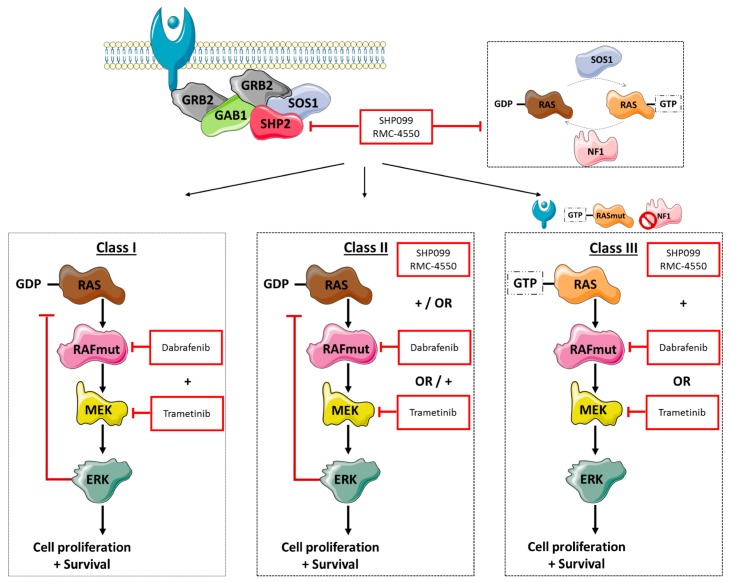
Our model of the BRAF mutation positive mitogen-activated protein kinase (MAPK) pathway signaling mechanisms. Activation of receptor tyrosine kinases (RTKs) leads to stimulation of SOS1 through GRB2 and SHP2, and consequently triggers RAS signaling; in contrast neurofibromin 1 (NF1) can inactivate RAS. RAS activation leads to rapidly accelerated fibrosarcoma (RAF) stimulation, and in turn MEK and ERK activation. A negative feedback loop can then inhibit RAS signaling. Class I BRAF mutations induce a strong activation of the RAF kinase, independent of RAS signaling. Combined RAF (dabrafenib) and MEK (trametinib) inhibition was shown to effectively lower MAPK signaling and yielded a 64% response rate in non-small-cell lung cancer (NSCLC) patients. Class II BRAF mutations have similar characteristics, inducing intermediate to high levels of RAF kinase activation, independent of RAS. In this study we have shown that dual RAF and MEK inhibition yielded synergistic results at median drug concentrations, while being antagonistic at either low or high concentrations. In addition, trametinib or dabrafenib treatment combined with SHP2 inhibition (SHP099 or RMC-4550) indicated synergistic interactions. The class III BRAF mutations often co-occur with RTK overexpression, RAS activating mutations, and/or NF1 loss-of-function mutations, indicating that cell growth depends on RAS signaling. For this reason, class III mutations are sensitive to single SHP2 inhibition, and combining trametinib or dabrafenib with SHP2 inhibitors showed strong synergy. RTKs: receptor tyrosine kinases.

Under physiological conditions, RTK—and consequently GRB, GAB1, and SHP2 signaling—control the activation of RAS proteins through SOS1 and NF1. Once activated, RAS can then lead to downstream signaling through RAF dimer formation and MEK and ERK activation. Although the MAPK pathway is tightly regulated, mutations in *BRAF*—with or without other co-alterations—can cause uncontrolled cellular proliferation and cell survival, as shown in Figure 5. At present, NSCLC patients harboring V600 *BRAF*—or class I—mutations can be effectively treated with a combination of MEK and BRAF inhibitors. However, there are currently no approved targeted treatments for the 50–80% of patients with non-V600 *BRAF* mutations. In this study, we confirm that complete exon sequencing with a cfDNA NGS panel enabled non-invasive detection of class I, II, III, and “other” *BRAF* mutations plus amplifications. Primary analysis of the SLLIP study focused on comparing standard-of-care tissue genomic testing and cfDNA NGS testing for the detection of guideline-recommended biomarkers. Of note, none of the four *BRAF* V600E mutations detected in plasma were identified via standard-of-care tissue genomic testing in the SLLIP study, highlighting the importance of liquid biopsies for mutation detection. In our patient cohort, approximately 80% of *BRAF* altered patients comprised non-V600 alterations, including class II, class III, *BRAF* amplifications, and “other” mutations that have not been classified yet. This preponderance of non-V600 *BRAF* alterations highlights the importance of developing new treatment options for patients harboring these mutations.

For effective use of targeted treatment, it is important to know which mutations are functionally relevant drivers of the tumor, and which mutations are passengers. Previous findings that class II *BRAF* mutations are oncogenic drivers of lung adenocarcinoma [14] are confirmed in our study, as the Guardant360 test sequences all 20 mutually exclusive oncogenic drivers in lung adenocarcinoma, and in most patients mutations in other known drivers did not co-occur with class II *BRAF* mutations. Of the four class III *BRAF* mutations, two *EGFR* mutations were co-occurring and only one of those *EGFR* mutations was found to be a driver mutation. Therefore, our results support that the class II and class III *BRAF* alterations are indeed driver mutations in lung adenocarcinoma. In the Class I *BRAF*-mutant cohort, additional *KRAS* mutations were found in two out of four patients. In one patient, the *BRAF* V600E was determined to be the driver mutation, and in the other patient, an additional *HRAS* mutation was found to be the driver. Other passenger mutations were highly heterogeneous among patients, and although the groups consist of small numbers of patients, we found a trend of higher *P53* alteration frequency in class II and III *BRAF*-mutant groups. Moreover, we hereby confirm that in the clinical setting, class III *BRAF* mutations coexist with *NF1* nonsense and missense mutations. 

Previously published results indicate that non-V600 *BRAF*-mutant patients tend to have improved PFS to platinum-based chemotherapy, compared to *BRAF* V600 mutation-positive patients [15]. In contrast, another study reported shorter PFS for class II and III BRAF-mutant patients on chemotherapy, compared to class I *BRAF*-mutant patients [16]. Although our small cohorts cannot be used to draw any conclusions, non-V600 BRAF-mutant patients in the SLLIP study tend to have improved PFS to platinum-based chemotherapy. In addition, we found that patients treated with immunotherapy—independent of *BRAF* mutation class—tend to have longer PFS than patients treated with targeted- or chemotherapy. Intriguingly, a recent study reported that MAPK alterations, including *BRAF* and *SHP2* mutations, were enriched in patients that responded to the PD-1 inhibitors nivolumab and pembrolizumab [17]. Since our patient cohort was small, further exploratory studies should be performed to validate these findings. 

It is now known that *BRAF*-mutation positive cancer is a heterogeneous disease, in which the location of the primary tumor may be less important than initially thought, and we hypothesize that focusing on specific alterations may be more important. Understanding the characteristics and divergent signaling properties of these different classes of mutations is critical for the development of effective therapeutic approaches. To this end, we explored the effect of dual treatment with RAF and MEK inhibitors on V600 and non-V600 *BRAF*-mutant cells, as shown in Figure 5. While in patients dual treatment yielded a 64% response rate and a median PFS of 10.9 months in *BRAF* V600 mutation-positive NSCLC, combined RAF/MEK treatment in the class I *BRAF*-mutant melanoma cell line (WM793) displayed additive to antagonistic effects. Class II *BRAF*-mutant (MDA-MB-231) cells were found to be sensitive to dual RAF/MEK treatment, only at median drug concentrations. In addition, class I and class II *BRAF*-mutant cells were intermediately sensitive and resistant, respectively, to single SHP2 inhibition, which can be explained by their RAS signaling independence [7]. When RAF/MEK inhibitors were combined with SHP2 inhibition in the class I and II *BRAF*-mutant and *BRAF* WT cell lines, we obtained moderate synergistic effects. However, the main effect was derived from single RAF/MEK inhibition or single SHP2 inhibition. In the class III *BRAF*-mutant cell line (H1666), we obtained antagonistic effects after combined RAF and MEK treatment. However, this cell line was sensitive to single SHP2 inhibition, due to the dependence on upstream RAS signaling. Cell viability experiments of combined RAF or MEK plus SHP2 inhibition in the class III *BRAF*-mutant cell line indicated clear synergistic effects, revealing a possible new treatment approach for this *BRAF*-mutant subtype. These results should be further exploited in vitro and in vivo, and if confirmed would warrant clinical development of class-specific therapy in *BRAF*-altered patients. 

## 4. Materials and Methods

### 4.1. Patients

This study provides a secondary analysis of the Spanish Lung Liquid vs. Invasive Biopsy Program (SLLIP) study, a multi-center prospective study (clinicaltrials.gov registration NCT03248089) designed to evaluate the Guardant360® NGS assay in cfDNA compared to standard-of-care tissue-based genomic testing for the detection of guideline-recommended biomarkers in previously untreated NSCLC patients. The study was approved by the Agencia Española de Medicamentos y Productos Sanitarios (AEMPS) and by the Ethics Committee of Investigación del Grupo Hospitalario Quirón (MedOPP125). Subjects were >18 years of age with a confirmed diagnosis of metastatic non-squamous NSCLC with no prior treatment for advanced NSCLC and an Eastern Collaborative Oncology Group (ECOG) performance status of 0–2. For this study, only patients that harbored BRAF alterations in baseline cfDNA analysis were included.

### 4.2. Procedures

Peripheral blood (20 mL) was collected in Streck^®^ Cell-free DNA Blood Collection Tubes prior to treatment and sent to Guardant Health, Inc. Over the course of the study, plasma samples were collected and analyzed at two weeks after starting first-line therapy, and during progression or at the end of study (EOS) using the Guardant360 assay. The Guardant360 test employs digital sequencing to completely sequence the critical exons in 73 genes, including all 18 exons in *BRAF*, and identifies all four major classes of alterations: single nucleotide variants (SNVs), indels, and selected fusions and copy number amplifications. *BRAF* copy number amplification is reported as absolute plasma copy number and is a result of tissue copy number amplification and the degree of tumor DNA shed into the circulation. *BRAF* plasma copy number is filtered for focal amplifications through comparison to three other genes (*EGFR*, *MET*, *CDK6*) that reside, with *BRAF*, on chromosome 7. Clinical data, including overall response rate (ORR) and PFS were collected for all patients. 

### 4.3. Data Analysis

Clinical data was used to explore the effect of BRAF class on PFS. The PFS in this study was defined as the time from the start of the first line treatment, to the date of progression or death. Patients who did not progress, neither die, were censored at the last visit date where their status was confirmed to be alive and without progressive disease (PD). Patients that did not start first-line treatment were excluded from analysis. Due to the small sample sizes, data analysis was considered to be exploratory.

### 4.4. Chemicals and Reagents

Human lung adenocarcinoma H1666 cells, harboring BRAF G466V (Class 3) mutation, human TNBC, MDA-MB-231 cells, harboring BRAF G464V (Class 2), and human melanoma WM793 cells, harboring BRAF V600E, were purchased from the American Type Culture Collection (ATCC). Human lung adenocarcinoma PC9 cells, harboring EGFR exon 19 deletion, but no BRAF mutations were provided by Mayumi Ono, (Kyushu University, Fukuoka, Japan). All cell lines were maintained in RPMI (Roswell Park Memorial Institute medium) 1640 supplemented with 1% Gibco penicillin/streptomycin/glutamine (Thermo Fisher Scientific, Waltham, MA, USA) and 10% Gibco fetal bovine serum (FBS; Thermo Fisher Scientific, Waltham, MA, USA) in a 5% CO_2_ 37 °C cell culture incubator and routinely evaluated for mycoplasma contamination. The MEK inhibitor trametinib, BRAF inhibitor dabrafenib, and both SHP2 inhibitors SHP099 and RMC-4550 were bought from Selleck Chemicals (Houston, TX, USA). Drugs were prepared in dimethylsulfoxide (DMSO) at a concentration of 10–100 mM stock solutions and stored at −20 °C. Further dilutions were made in culture medium to final concentration before use. Antibodies rabbit anti-ERK (#9102) and rabbit anti-phospho-ERK (T202/Y204; #9101) were purchased from Cell Signaling (Leiden, The Netherlands) and diluted 1:1000. Mouse anti-Beta-actin (#A5441) was purchased from Sigma Aldrich/Merck (Darmstadt, Germany) and diluted 1:5000. Secondary horseradish peroxidase (HRP)-linked goat anti-rabbit (from donkey, NA934-1ML) and HRP-linked goat anti-mouse (from sheep; NA931-1ML) were purchased from GE Healthcare Life Sciences (Amersham, UK) and diluted 1:5000.

### 4.5. Cell Viability Assay

Cells were seeded in 96-well plates at the following densities: 1.0 × 10^3^ (PC9), 1.5 × 10^3^ (WM793), 2.5 × 10^3^ (H1666), and 1.5 × 10^3^ (MDA-MB-231), and incubated for 24 h. Cell viability was assessed using the 3-[4,5-dimethylthiazol-2-yl]-2,5-diphenyltetrazolium bromide (MTT) assay (Sigma Aldrich, St. Louis, MO, USA), after treatment with serial drug dilutions. For the half maximal inhibitory concentration (IC_50_) determination, MTT viability assays were performed using SHP099 (all cell lines) and RMC-4550 (H1666 and MDA-MB-231) doses ranging from 0–100 μM and trametinib doses ranging from 0–2 μM. For dabrafenib, doses ranged from 0–500 μM for MDA-MB-231, PC9 and WM793 and 0–600 μM for H1666. 

To determine the combined effect of trametinib and dabrafenib on cell viability, cells were treated with trametinib doses ranging from 0 to 2 μM, and dabrafenib doses ranging from 0 to 500 μM (MDA-MB-231, PC9, and WM793) or 0–600 μM (H1666), or with the combination of both. In addition, cells were treated with trametinib doses ranging from 0 to 2 μM, and SHP099 or RMC-4550 doses ranging from 0 to 100 μM, or with a combination of trametinib plus SHP099 or RMC-4550. Finally, cell lines were treated with dabrafenib doses from 0 to 500 μM (MDA-MB-231, PC9, and WM793) or 0–600 μM (H1666), and SHP099 or RMC-4550 doses ranging from 0 to 100 μM, or with a combination of dabrafenib plus SHP099 or RMC-4550. After 72 h of treatment incubation, 0.5 mg/ml of MTT was added to the medium in the wells for 2 h at 37 °C and formazan crystals in viable cells were solubilized with 100 μl DMSO and spectrophotometrically quantified using a microplate reader (Varioskan Flash; Thermo Fisher Scientific, Waltham, MA, USA) at 565 nm of absorbance. Fractional survival was then calculated as a percentage to control cells. Data of combined drug effects were subsequently analyzed by the Chou and Talalay method [18,19]. Combination Index (CoI) values <1, =1, and >1 indicated synergism, additive effect, and antagonism, respectively. All experiments were performed in biological triplicates. 

### 4.6. Western Blotting

For immunoblotting experiments, cells were seeded in T25 flasks (Sarstedt, Newton, NC, USA). Cell amounts were 0.5 million per flask (PC9, MDA-MB-231, and H1666), 0.75 million per flask (WM793), after 24 h, cells were either treated with increasing doses of SHP099 (5, 10, and 50 μM), or with increasing doses of trametinib (10, 20, and 50 nM). Untreated cells received an equivalent dose of vehicle (DMSO). After 24 h, cells were washed with cold PBS and re-suspended in ice-cold radioimmunoprecipitation assay (RIPA) buffer (50 mM tris-hydrochloric acid in pH 7.4, 1% Nonidet P-40, 0.5% sodium deoxycholate, 0.1% sodium dodecyl sulfate [SDS], 150 mM sodium chloride, 1 mM ethylenediaminetetraacetic acid, 1 mM sodium vanadate, and 50 mM sodium fluoride) containing protease inhibitor mixture (Roche Applied Science, Penzberg, Germany). Following cell lysis by sonication and centrifugation at 18,620× *g* for 10 min at 4 °C, the resulting supernatant was collected as the total cell lysate. Briefly, the lysates containing 30 μg proteins were electrophoresed on 10% SDS-polyacrylamide gels (Life Technologies, Carlsbad, CA, USA) and transferred to polyvinylidene difluoride (PVDF) membranes (Bio-Rad Laboratories Inc., Hercules, CA, USA). Membranes were blocked in Odyssey blocking buffer (Li-Cor Biosciences, Lincoln, NE, USA). All target proteins were immunoblotted with appropriate primary and horseradish peroxidase (HRP)-conjugated secondary antibodies. Chemiluminescent (HRP-conjugated) bands were detected in a ChemiDoc MP Imaging System (Bio-Rad Laboratories Inc.). β-actin was used as an internal control to confirm equal gel loading. Experiments were performed in biological triplicates with similar results, and representative blots were shown.

## 5. Conclusions

Using an NGS assay on plasma-derived cfDNA provides a non-invasive approach of identifying significant numbers of class II and III *BRAF* mutation-positive patients, which are potential candidates for enrollment in combination targeted therapy trials that exploit the synergistic effects of inhibition of SHP2 and BRAF as elucidated here.

## Figures and Tables

**Table 1:** Baseline clinical characteristics for all BRAF-alteration positive patients (*n* = 22).

Baseline Patient Clinical Characteristics			Total (*n* = 22)		
**Gender**			**Histology**		
Female	*n* (%)	9 (40.9)	Adenocarcinoma	*n* (%)	21 (95.5)
Male	*n* (%)	13 (59.1)	Large cell carcinoma	*n* (%)	1 (4.5)
**Age (years)**			**Treatment**		
	Mean (SD)	63.0 (11.2)	Chemotherapy	*n* (%)	10 (45.5)
	Median (Q1, Q3)	62.0 (56.0, 73.0)	Immuno + Chemotherapy	*n* (%)	3 (13.6)
	Min, Max	39.0, 80.0	Immunotherapy	*n* (%)	1 (4.6)
	Mean CI 95%	(57.6, 68.4)	Targeted treatment	*n* (%)	5 (22.7)
			No treatment	*n* (%)	3 (13.6)
**Race**			**Stage**		
Caucasian	*n* (%)	22 (100.0)	IVB	*n* (%)	22 (100.00)
**Smoking Status**			**ECOG PS**		
Ex-smoker	*n* (%)	4 (18.2)	0	*n* (%)	6 (27.3)
Non-smoker	*n* (%)	5 (22.7)	1	*n* (%)	14 (63.6)
Smoker	*n* (%)	13 (59.1)	2	*n* (%)	2 (9.1)

ECOG PS: Eastern Cooperative Oncology Group performance status.

**Table 2:** Cell line characteristics.

Cell Lines	Characteristics			IC50s			
	Tumor Type	BRAF Mutation	BRAF Class	Dabrafenib	Trametinib	SHP099	RMC-4550
**WM793**	Melanoma	V600E	Class 1	0.05 μM	0.0003 μM	30.2 μM	-
**MDA-MB-231**	TNBC	G464V	Class 2	17.0 μM	0.0210 μM	69.4 μM	63.0 μM
**H1666**	Lung ADC	G466V	Class 3	90.4 μM	0.0240 μM	9.8 μM	3.1 μM
**PC9**	Lung ADC	WT	WT	55.0 μM	>2.0 μM	26.8 μM	-

TNBC: triple negative breast cancer; ADC: adenocarcinoma; WT: wild type.

## Supplementary Materials

The following are available online at https://www.mdpi.com/2072-6694/11/9/1381/s1, Figure S1: MTT viability assays with IC_50s_ of SHP2 inhibitors SHP099 and RMC-4550 in the class II and class III cell lines, Figure S2: MTT cell viability assays combining either a BRAF (dabrafenib) or a MEK inhibitor (trametinib) with an SHP2 inhibitor (RMC-4550) in the class II and class III cell lines.
