# Supplementary material for: BRAF Mutations Classes I, II, and III in NSCLC Patients Included in the SLLIP Trial: The Need for a New Pre-Clinical Treatment Rationale
Source: Cancers (Basel). 2019 Sep 17;11(9):1381. doi: 10.3390/cancers11091381 (PMC6770188; doi:10.3390/cancers11091381)
Supplement: Supplementary file 1 [file cancers-11-01381-s001.pdf]

Supplementary Material: *BRAF* Mutations Classes I, II, and III in NSCLC Patients Included in the SLLIP Trial: The Need for a New Pre-Clinical Treatment Rationale

Jillian Wilhelmina Paulina Bracht, Niki Karachaliou, Trever Bivona, Richard B. Lanman, Iris Faull, Rebecca J. Nagy, Ana Drozdowskyj, Jordi Berenguer, Manuel Fernandez-Bruno, Miguel Angel Molina and Rafael Rosell

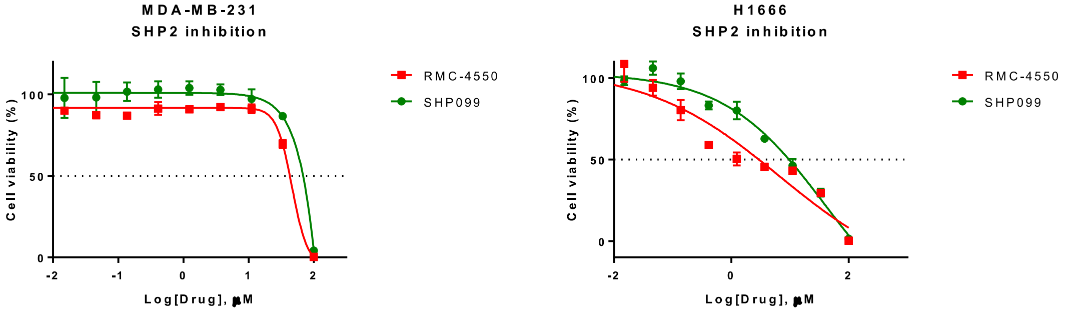

SHP099: 69.4 µM
RMC-4550: 63.0 µM

SHP099: 9.8 µM
RMC-4550: 3.1 µM

Figure 1. MTT cell viability assays were performed in the class II (MDA-MB-231) and class III (H1666) *BRAF*-mutant cell lines, to compare the IC_{50s} of two distinct SHP2 inhibitors: SHP099 and RMC-4550. IC_{50}: half maximal inhibitory concentration.

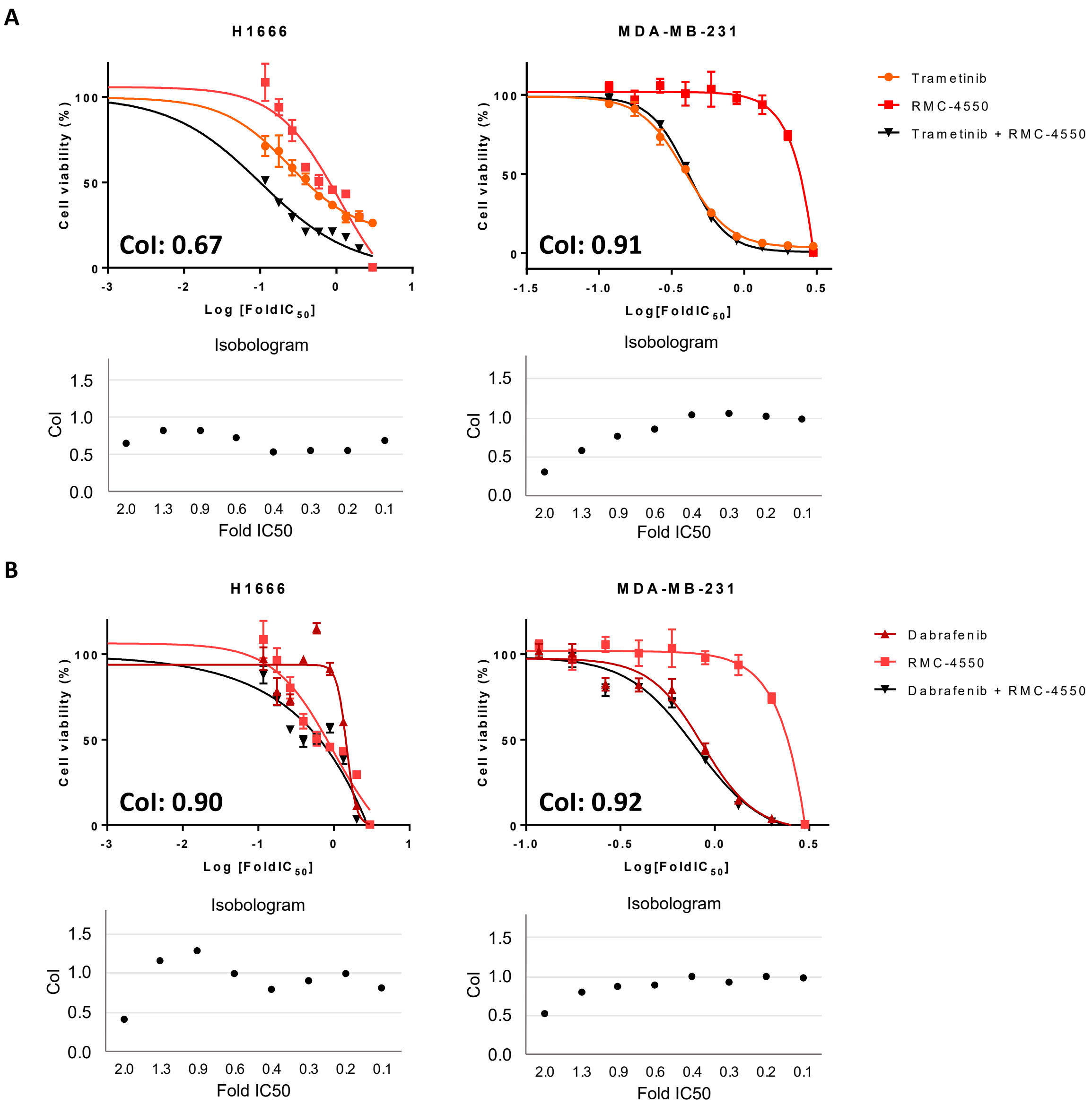

Figure S2. MTT cell viability assays were performed in the class II (MDA-MB-231) and class III (H1666) *BRAF*-mutant cell lines, to compare the effect of single MEK (trametinib) and single SHP2 (RMC-4550) treatment, or combined treatment on cell viability (**A**) and to compare the effect of single BRAF (dabrafenib) and single SHP2 (RMC-4550) treatment, or combined treatment on cell viability (**B**). The isobolograms depict combination index (CoI) values at each drug concentration, calculated based on the Chou and Talalay method. Average CoIs are depicted in the graph, and CoI values <1, = 1, and >1 indicate synergism, additive effect and antagonism, respectively. Experiments were performed in biological triplicates with similar results, and representative graphs are shown.

© 2019 by the authors. Licensee MDPI, Basel, Switzerland. This article is an open access article distributed under the terms and conditions of the Creative Commons Attribution (CC BY) license (http://creativecommons.org/licenses/by/4.0/).
